# Supplementary material for: Anti-Bacterial Effect of CpG-DNA Involves Enhancement of the Complement Systems
Source: Int J Mol Sci. 2019 Jul 10;20(14):3397. doi: 10.3390/ijms20143397 (PMC6678731; doi:10.3390/ijms20143397)
Supplement: Supplementary file 1 [file ijms-20-03397-s001.pdf]

Anti-Bacterial Effect of CpG-DNA Involves Enhancement of the Complement Systems

Te Ha Kim, Joongwon Park, Dongbum Kim, Avishekh Gautam, Madhav Akauliya, Jinsoo Kim, Hanseul Lee, Sangkyu Park, Younghee Lee, Hyung-Joo Kwon

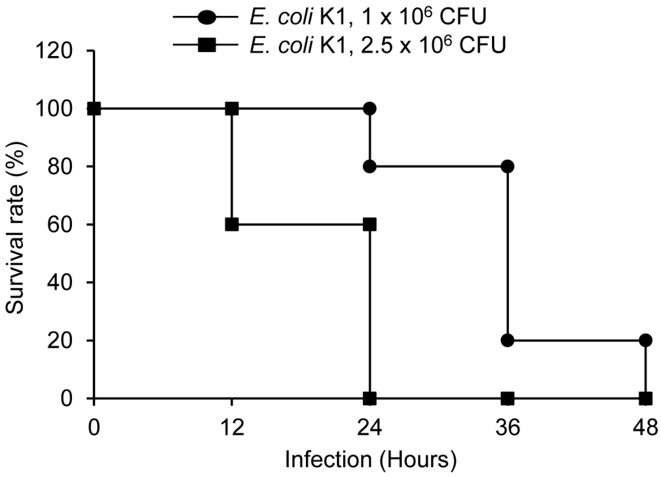

Supplementary Figure S1. Dose-dependent survival rate of the mice after *E. coli* K1 infection. BALB/c mice (n=5/group) were i.p. injected with the indicated CFUs of *E. coli* K1, and the survival of the mice was monitored for 48 h.

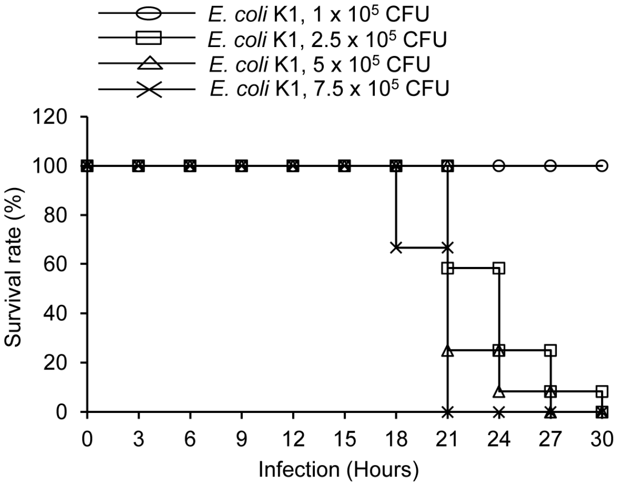

Supplementary Figure S2. Effect of the CVF injection on the survival rate of the *E. coli* K1-infected mice. BALB/c mice (n=12/group) were i.p. injected with CVF (30 μg/mouse). After 6 h, the mice were i.p. injected with the indicated CFUs of *E. coli* K1, and the survival of the mice was monitored for 30 h.
